# Factors associated with subjective burden among informal caregivers of home-dwelling people with dementia: a cross-sectional study

**DOI:** 10.1186/s12877-023-04358-3

**Published:** 2023-10-10

**Authors:** Gunn Steinsheim, Wenche Malmedal, Turid Follestad, Bonnie Olsen, Susan Saga

**Affiliations:** 1 Department of Public Health and Nursing, Norwegian University of Science and Technology (NTNU), Postbox 8905, Trondheim, N-7491 Norway; 2 Department of Clinical and Molecular Medicine, Norwegian University of Science and Technology (NTNU), Trondheim, Norway; 3 Clinical Research Unit Central Norway, St. Olavs Hospital, Trondheim, Norway; 4 Department of Family Medicine, Keck School of Medicine, University of Southern California, Los Angeles, USA

**Keywords:** Caregiver burden, Cross-sectional studies, Dementia, Family caregivers, Norway, Regression analysis, Surveys and questionnaires

## Abstract

**Background:**

It is estimated that more than 57 million people have dementia worldwide, and it is one of the leading causes of care dependency in old age. Relatives and other informal caregivers are the most important support for individuals with dementia, but caring for a loved one with dementia may burden the caregiver. Caregiver burden may have adverse outcomes for both the informal caregiver and the care recipient, including decreased quality of life. Caregiver burden is associated with several factors concerning the informal caregiver, the care recipient, and relational and other contextual factors. The aim of this study was to explore which factors are associated with informal caregivers’ subjective burden when caring for individuals living at home with dementia.

**Methods:**

This study was a cross-sectional survey among informal caregivers of home-dwelling individuals with dementia in all five geographical regions of Norway. There were 540 informal caregivers who participated, 415 of whom were included in the regression analyses. Caregivers’ subjective burden was assessed with the Relatives’ Stress Scale. Covariates included were classified into four levels: individual (twofold: informal caregiver and person with dementia), relational, community, and time. Linear multivariable regression analyses were used to identify associations between subjective burden and included factors.

**Results:**

Several covariates were statistically significantly associated with subjective burden at the four levels. These covariates included self-rated health, mental distress, age, coping through resignation and denial, emotional and instrumental support, substance use, and humor at the informal caregiver level; behavioral and psychological symptoms of dementia, dementia severity, and degree of disability at the care recipient level; the extent of care, being the primary caregiver, and previous relationship satisfaction at the relational context level; and informal caregivers spending time with friends, leisure activities, social restriction, and knowledge of available health services at the community context level.

**Conclusions:**

Informal caregivers’ mental distress and care recipients’ neuropsychiatric symptoms were the factors with the strongest association with subjective burden.

**Supplementary Information:**

The online version contains supplementary material available at 10.1186/s12877-023-04358-3.

## Introduction

It is estimated that more than 57 million people have dementia worldwide and that the number will almost triple by 2050 [1]. As one of the leading causes of care dependency in old age, dementia considerably impacts individuals with dementia, their families, and society [2]. Relatives and other informal caregivers are the most important support for people with dementia. The global informal care cost of dementia is approximately 651.4 billion US$, which accounts for nearly half the total cost of dementia (ibid.). In Europe, informal caregivers spend about 7.4 h a day assisting with activities of daily living (ADLs) and supervision per person with dementia [2].

In Norway, 14.7% of people aged 70 and above are estimated to have dementia [3]. Furthermore, the proportion of the population aged 70 and up is predicted to rise from 13% today to 22.4% by 2060 [4], which will result in tremendous growth in people with dementia. Since 2007, Norway has had national dementia plans with strategies to improve services for individuals with dementia and their families [5–7]. Support and services for people with dementia and their informal caregivers in Norway include home nursing services, practical homecare services, adult daycare services, and dementia education or peer support groups for people with dementia or their informal caregivers [8]. Health services are mainly financed by public spending (85%) with some level of personal funding [9], but home nursing services are free of charge.

Informal caregivers are people who regularly provide unpaid care to a family member or friend with a health condition or disability [10]. In a large Norwegian study involving more than 2000 persons with dementia, Vossius et al. found that nine out of ten individuals with dementia receive informal help and care from relatives or friends [11]. Further, they estimated that caregivers provide an average of 60 to 80 h of care monthly at the time of diagnosis. Just before admission to a nursing home, the number of hours of informal care per month is doubled [11]. Although there are many positive aspects of caring for a family member or friend with dementia, such as a sense of emotional reward and personal growth [12], it may also be a burden to the caregiver. Caregiver burden is related to the strain and distress informal caregivers may experience as a result of their care. Caregiver burden is a complex multi-dimensional concept [13] and in some research, a distinction is made between objective burden —related to events and activities caused by caregiving—and subjective burden—related to the informal caregiver’s attitudes and emotions due to caregiving [14]. According to Wolfs et al. [13] objective burden relates to the time commitment and the nature and quantity of tasks, impacting care-related costs, while subjective caregiver burden reflects the caregiver’s personal experience. The authors describe that subjective caregiver burden is shaped by care recipient characteristics such as behavioral problems or cognitive decline, as well as aspects related to the caregiver, such as gender and age, along with social factors like the presence of social support and family assistance [13].

Caregiver burden may have adverse outcomes for both the informal caregiver and the care recipient. Some potential negative outcomes for the care recipient are institutionalization, elder abuse, worsening of neuropsychiatric symptoms of dementia and cognitive impairment, and decreased quality of life [15]. For informal caregivers, the physical, psychological, social, and financial aspects of caregiving may have a negative effect on their physical and mental health [2]. Informal caregivers tend to experience more stress, more depression, lower self-efficacy, and lower subjective well-being compared to non-caregivers, and the negative outcomes are more pronounced among those caring for someone with dementia [16].

Caregiver burden is associated with several factors concerning the informal caregiver, the care recipient, and relational and other contextual factors. Factors related to informal caregivers include physical and mental health [17], such as anxiety and depression [18]. Informal caregivers’ coping strategies have also been found to be associated with or to mediate caregiver burden [19–21]. Coping is a way of responding to a perceived threat [22], which can either limit or increase the capacity to endure the threat [23]. Factors related to the care recipients include neuropsychiatric symptoms, disability [17, 24], and cognitive decline [25, 26]. Relational factors such as kinship, cohabitation [17], and a poor premorbid relationship [27] are also associated with caregiver burden. Social support has been found to moderate the association between care recipient cognitive function and caregiver burden [28], while informal caregivers’ social isolation has been associated with increased caregiver burden [27]. In addition, various interventions have been developed to lower burden and increase coping for informal caregivers of people with dementia. However, multicomponent interventions combining individual training or counseling and group sessions [29] and interventions focusing on increasing knowledge of dementia and coping skills [30] have only demonstrated small to medium effects on reducing caregiver burden.

In the present study, we build on previous studies investigating factors associated with caregiver burden. Although several previous studies have explored factors associated with caregiver burden, there are still inconsistencies and uncertainties including sex and gender differences [31] and the effect of caregiver age on burden [17]. Many studies have used small sample sizes or controlled for only a few covariates. Therefore, we seek a large, geographically and demographically diverse sample, include a large number of relevant factors in the survey, and use validated measures for key concepts. We adopted the structure of a contextual theory of elder abuse [32] and Bronfenbrenner’s chrono level, which involves change over time [33]. The contextual theory of elder abuse was developed to address the complexities of elder abuse encompassing four contexts: the individual, the relational, the community, and the societal [32]. The theory builds upon Bronfenbrenner’s ecological model [33]. We chose this framework because, like elder abuse, caregiver burden is a multifaceted concept influenced by factors within individuals, their relationships, and their environment. Thus, we classified covariates into four levels: individual, relational, community, and time. This theoretical framework emphasizes individual and contextual factors, which in this study might be associated with informal caregivers’ subjective burden. Using data from a cross-sectional study of informal caregivers in Norway, we aimed to explore which factors are associated with informal caregivers’ subjective burden when caring for individuals living at home with dementia.

## Methods

### Study design

This is a cross-sectional study among informal caregivers of home-dwelling individuals with dementia in Norway and is part of a more extensive study exploring caregiver burden and elder abuse among home-dwelling individuals with dementia [34]. The study has been approved by the Regional Committee for Medical Research Ethics Central Norway (#153,444), and informed consent was obtained from all study participants.

### Data collection

The study recruited participants from all five geographical regions of Norway from May to December 2021. We collected data through anonymous self-reported pen-and-paper questionnaires. In 2021, there were still COVID-19 restrictions possibly affecting people with dementia and their informal caregivers. National restrictions to prevent the spread of COVID-19 shifted throughout 2021, beginning with stricter regulations in March and gradually reducing through the end of September [9]. Because of local outbreaks, local restrictions also varied. Social distancing regulations impacted services for individuals with dementia, especially daycare activities that provided social interaction.

A convenience sample of informal caregivers was recruited using three strategies: (1) direct invitation through The Norwegian Registry of Persons Assessed for Cognitive Symptoms, (2) referral sampling through municipal health services and local volunteer dementia associations, and (3) self-enrollment through social media and magazines. The participants received the questionnaire by mail or directly through contact persons in the municipal health services and local volunteer dementia association, and completed and returned the questionnaires anonymously in a stamped return envelope. Details of recruitment and participation have been described previously [35]. Eligibility criteria for participants were: (1) informal caregiver of a person with dementia currently living at home, (2) aged > 18 years, (3) spouse, cohabitant, partner, relative, or acquaintance who had contact with the person with dementia at least once a week, (4) preferably the primary caregiver. If the primary caregiver could not participate, another caregiver could answer if they met the other inclusion criteria. Participants were primarily identified and recruited through registries, health care service providers, or organizations specializing in dementia care, and their family member or friend had a confirmed diagnosis of dementia. However, for some, the diagnostic process was still ongoing to determine the specific type of dementia.

### Measurements

The questionnaire for this survey was developed through collaborations with user organizations, interviews with caregivers, and a small pilot study. Amendments were made after feedback, and details on the development can be found in a previous publication [36].

*Caregiver burden* was measured using the Relatives’ Stress Scale (RSS) [37]. The RSS consists of 15 items measuring different aspects designed to measure the strain relatives experience when caring for an older person. The items concern how often the informal caregivers feel they can no longer cope, feel they need a break, get depressed by the situation, feel there will be no end to the problems, have been prevented from having visitors, feel embarrassed by the care recipient, get cross and angry with the care recipient, and feel frustrated with the care recipient. Further, the questions concern the extent to which the informal caregivers experience the following: that their standard of living has been reduced, that it is difficult to get away on holiday, that their own health has suffered, that their social life has been affected, that the household routines have been disrupted, that they worry about accidents happening to the care recipient, and that their sleep is interrupted by the care recipient. The items are measured on a scale from 0 (“Never/not at all”) to 4 (“Always/considerably”). This provides a possible sum score ranging from 0 to 60, with a higher score indicating a higher burden. The Norwegian version of the RSS is widely used in clinical practice to measure caregiver burden, and it has been validated by Ulstein et al. [38]. In the present study, the RSS showed good internal consistency (Cronbach’s alpha = 0.92).

*Socio-economic aspects* related to the informal caregiver and their care recipient, such as age, gender, and informal caregiver’s educational level, were collected. The informal caregiver’s perceived economic status was measured by the question, “How easy or difficult is it for your household to get the money to suffice on a daily basis, with your income?” on a six-point rating scale from 1 (“Very difficult”) to 6 (“Very easy”), along with a “Do not know” option. The values 1–3 were recoded to the category “Difficult” and 4–6 to the category “Easy” before analyses. “Do not know” was kept as a separate category. Informal caregiver leisure activity was measured by four questions concerning physical, cultural, social, and religious activities on a scale from 0 (“Never”) to 5 (“Daily”). The four questions were merged into a sum score with a possible range from 0 to 20, where a higher score indicates more leisure activity. Informal caregiver’s spending time with friends was measured by the question, “How often are you with good friends?” Care recipient activity was measured by two questions concerning social and cultural activity, which were merged into a binary variable with the categories “No activity” and “Any activity.”

*Informal caregivers’ coping strategies* were measured with a Norwegian version [39] of the Brief COPE [40], which consists of 28 items measuring 14 different coping reactions. We used the situational retrospective format, and the informal caregivers were asked to consider what they usually do when they experience stress related to caregiving. The items are scored from 0 (“I haven’t been doing this at all”) to 3 (“I’ve been doing this a lot”). Due to inconsistencies with the factor structure of the brief COPE [41], we performed an exploratory factor analysis with principal component factoring (PCF), resulting in an eight-factor solution (see supplementary). We selected five factors for further analyses based on the result of the factor analyses and previous studies. The variable “Giving up” consists of items 3, 6, 8, and 16, which measure strategies related to giving up on dealing with the situation and denial. The variable “Support” consists of items 5, 10, 15, and 23, which measure emotional and instrumental support use. The three remaining variables were all composed of two items that corresponded with the original scale’s “Substance use,” “Humor,” and “Religion.” The 4-item factors had a possible sum score from 0 to 12, while the 2-item factors ranged from 0 to 6, with a higher score indicating more use of the specific coping strategy. All factors showed acceptable to excellent internal consistency (Cronbach’s alpha = 0.76–0.91).

*Informal caregiver health* was measured with one item and a screening tool. Self-rated health was measured with a single item, where informal caregivers rated their health on a five-point scale from 1 (“Poor”) to 5 (“Very good”). Mental health or distress was measured with the four-item version of the Patient Health Questionnaire (PHQ4) [42], which is a short screening tool for anxiety and depression. Participants reported how often they have been bothered by each of the four problems in the past two weeks, and items were scored on a scale from 0 (“Not at all”) to 3 (“Nearly every day”) which results in a sum score ranging from 0 to 12. According to Kroenke et al. [42], one can categorize the sum scores as normal (0 − 2), mild (3–5), moderate (6–8), and severe (9 − 12) psychiatric distress. The Norwegian version has shown good psychometric properties [43, 44].

*Neuropsychiatric symptoms* in individuals with dementia was measured by the Neuropsychiatric Inventory Questionnaire (NPI-Q) [45]. The NPI-Q consists of 12 items related to different neuropsychiatric symptoms, measuring if the symptom has been present in the past month and the severity of the symptom on a three-point rating scale of 1 (“Mild”), 2 (“Moderate”), and 3 (“Severe”). This results in a sum score ranging from 0 to 36, with a higher score indicating a more severe symptom burden. The Norwegian NPI-Q is a valid instrument for assessing the total symptom burden of neuropsychiatric symptoms [46].

*Dementia severity* was measured using the short form of the Informant Questionnaire on Cognitive Decline in the Elderly (IQCODE) [47, 48]. The scale consists of 16 items and is used as an informant screening test for dementia as a part of the basic dementia assessment in Norway. Informal caregivers were asked how the person with dementia was before they developed symptoms of dementia compared to the present situation. Items were scored on a five-point scale ranging from 1 (“Much improved”) to 5 (“Much worse”). A mean score was calculated if at least 80% of the items were non-missing, in accordance with Ringdal [49].

*Care recipient disability* was measured by the Rapid Disability Rating Scale 2 (RDRS2) [50]. This scale comprises items grouped into three domains: degree of assistance with activities of daily living, physical disabilities, and special problems. The items were ranked based on the person’s assistance need or disability on a scale from 1 (“None”) to 4 (“Total”). We excluded the special problems domain because of the overlap with items in the NPI-Q, resulting in a possible range between 18 (no disability) and 72 (severe disability).

*Social restriction* was measured by the Modified Social Restriction Scale [51]. This consisted of two questions addressing whether the informal caregiver had anyone else to care for the person with dementia if he/she got sick or needed a break from the caregiving role. The questions were rated on a three-point scale: 0 (“Yes, it would be easy to find someone”), 1 (“Yes, I could find someone, but it won’t be easy”), and 2 (“No, there is no one else”). The total sum score ranged from 0 to 4, with a higher score representing more social restriction.

*Previous relationship satisfaction* was measured by adapting a partnership satisfaction index used in the Norwegian Life Course, Ageing, and Generations panel study [52]. The index consists of five questions. We adapted the instructions and questions to fit the study setting and reduced the rating scale from a 0–10 scale to a 0–5 scale to reduce participant strain. The participants were instructed to think about their relationship with the care recipient prior to the dementia diagnosis. The participants were asked to rate four statements: (1) “We agreed about what is important in life,” (2) “We often had conflicts,” (3) “He/she often criticized me,” and (4) “He/she understood me when I had problems.” Participants rated each statement on a scale from 0 (“Strongly disagree”) to 5 (“Strongly agree”). Participants also responded to an overall satisfaction question, rating from 0 (“Not satisfied at all”) to 5 (“Very satisfied”). The scores from questions 2 and 3 were reversed before calculating a mean index score.

### Statistical analysis

Descriptive statistics are presented as means and standard deviations (SD) for continuous variables and frequencies and proportions for categorical variables. We performed factor analyses using principal component factoring (PCF) and calculated Cronbach’s alpha for scales.

We performed univariable and multivariable linear regression to explore the association between subjective burden (RSS) and the covariates. Linearity between the RSS and each continuous covariate in the univariable regression analyses was assessed by inspecting lowess (locally weighted estimated scatterplot smoothing) plots. Lowess is a nonparametric method that uses regression to fit a smooth curve for a series of data through a scatterplot [53]. For the multivariable regression analyses, we used Pearson correlation to check correlations between covariates regarding multicollinearity. We used visual inspection of a histogram and Q-Q plot for standardized residuals from the multivariable regression model to check for the assumption of a normally distributed error term (see Supplementary 2). To check for a constant variance of the error term, we inspected a scatterplot of standardized residuals versus predicted subjective burden (see Supplementary 2) and the Breusch–Pagan test for heteroskedasticity. No replacements were made for missing data, so listwise deletions were applied in the analyses. To examine whether deleted cases differ from complete cases, t-tests were used to compare the means of complete cases and deleted cases for each continuous variable. To investigate the relative importance of the covariates, we calculated standardized regression coefficients and squared semi-partial correlations in addition to inspecting the unstandardized regression coefficients [53]. The standardized coefficients show the mean standard deviation change in the outcome variable for a one standard deviation increase in the covariate. The squared semi-partial correlations reflect the covariate’s unique contribution to the outcome variable and may be interpreted as the decrease in R^2^ if the variable is removed from the model (ibid.). The significance level was set to 0.05. All statistical analyses were performed using Stata version 17 [54].

## Results

### Participant and descriptive data

A total of 540 informal caregivers from all parts of Norway participated in the study. The informal caregivers’ subjective burden (RSS) ranged from 0 to 60, with both a mean and median of 25 (Table 1). The informal caregivers were mostly female, in their 60 or 70s, currently not working, and living with the care recipient, and a vast majority were the primary caregiver of the care recipient. Among the informal caregivers, 10.6% experienced moderate to severe mental distress (PHQ4 score ≥ 6). The majority of care recipients were in their 70 or 80 s and had a mean disability score (RDRS2) of 39.8 and a mean dementia severity (IQCODE) score of 4.5.

**Table 1:** Descriptive characteristics of informal caregivers, their care recipients with dementia, and their relationship (N = 540)

	Range	Mean (SD)	n (%)	α*	Missing
**Individual context, informal caregiver**					
Burden - RSS	0–60	25.0 (11.7)		0.92	3.9%
Gender: Female			363 (68.9%)		1.5%
Edu.: High school or lower			307 (56.9%)		1.3%
University or higher			226 (43.1%)		
Employment: Full-time (incl. studies)			145 (28.5%)		2.0%
Part-time (incl. studies)			50 (9.6%)		
Not working			334 (62.0%)		
Economic status: Difficult			47 (8.5%)		1.3%
Easy			480 (90.2%)		
Do not know			6 (1.2%)		
Age	21–93	67.4 (11.8)			1.5%
Self-rated health	1–5	3.6 (0.9)			1.3%
Mental distress - PHQ4	0–12	2.8 (2.5)		0.86	2.2%
Coping strategies - brief COPE:					
Giving up	0–12	1.6 (2.1)		0.76	1.9%
Support	0–12	5.9 (2.6)		0.83	1.3%
Humor	0–6	1.5 (1.4)		0.76	1.7%
Religion	0–6	0.7(1.4)		0.91	0.9%
Substance use	0–6	0.3 (0.8)		0.89	0.6%
**Individual context, care recipient**					
Gender: Female			257 (48.6%)		0.7%
Age	53–99	78.9 (7.8)			0.7%
Neuropsychiatric symptoms - NPIQ	0–33	8.7 (6.1)		0.81	8.0%
Dementia severity - IQCODE	2.9-5	4.5 (0.5)		0.92	0.2%
Disability - RDRS2	18–68	39.8 (9.9)		0.90	3.7%
**Relational context**					
ICG primary caregiver: Yes			496 (93.3%)		1.3%
Relation to CR:					0.7%
Spouse			338 (63.1%)		
Child or other			198 (36.9%)		
Cohabiting: Yes			348 (63.6%)		0.9%
ICG previous relationship satisfaction	0–5	3.7 (1.0)		0.77	2.2%
Extent of care: Once a week or less			41 (7.9%)		1.3%
2–3 times a week			80 (15.5%)		
4–6 times a week			70 (12.6%)		
Every day			342 (64.0%)		
**Community context**					
ICG with friends: Weekly			193 (36.8%)		1.1%
Monthly			180 (33.9%)		
Infrequent			161 (29.3%)		
ICG leisure activity	0–14	6.8 (3.2)		0.55	2.2%
CR activity: No activity			112 (20.5%)		1.7%
Any activity			419 (79.5%)		
ICG knowledge of services	1–5	3.5 (0.9)			0.6%
ICG social restriction	0–4	1.7 (1.4)		0.87	1.9%
**Time context**					
Duration of care: 0-2y			181 (33.5%)		0.9%
3-5y			261 (49.0%)		
6y or more			93 (17.5%)		

*Cronbach’s alpha. ICG = informal caregiver, edu. = educational level, PHQ4 = Patient Health Questionnaire 4, CR = care recipient, NPIQ = Neuropsychiatric Inventory Questionnaire, IQCODE = Informant Questionnaire on Cognitive Decline in the Elderly, RDRS2 = Rapid Disability Rating Scale 2

### Associations with informal caregiver burden

Results from univariable and multivariable linear regression examining associations between informal caregiver subjective burden (RSS) and covariates can be found in Table 2. Missing data across the included variables resulted in 415 complete cases in the multivariable linear regression analyses, equivalent to 23.1% missing data due to listwise deletion. T-tests showed no statistically significant difference between means of complete cases and listwise deleted cases for the RSS, NPIQ, RDRS2, and PHQ4. The variables “Cohabiting” and “Relationship” were strongly correlated (r = 0.95) and caused multicollinearity in the multivariable model. If both were included, the direction of association with subjective burden for the relationship variable was changed, and the association was no longer statistically significant. Thus, only the cohabiting variable was included in the multivariable model.

**Table 2:** Results from linear regression analyses for caregiver burden (RSS) (N = 415)

	Univariable (model 1)				Multivariable (model 2)				
	Coef.	[95% CI]	p		Coef.	[95% CI]	p	Std.β	sr2
**Individual context, informal caregiver**									
Gender: Male (ref. Female)	-3.9	[-6.2, -1.6]	0.001		0.0	[-1.4, 1.3]	0.955	0.00	0.000
Edu.: University (ref. High school or lower)	-1.5	[-3.7, 0.7]	0.179		1.1	[-0.2, 2.4]	0.109	0.05	0.002
Employment (ref. Full-time incl. studies)									
Part-time incl. studies	2.5	[-1.5, 6.6]	0.222		-1.2	[-3.6, 1.2]	0.315	-0.03	0.001
Not working	3.2	[0.8, 5.7]	0.010		-0.3	[-2.2, 1.6]	0.742	-0.01	0.000
Economy (ref. Difficult)									
Easy	-9.2	[-13.4, -5.1]	< 0.001		2.4	[-0.1, 4.8]	0.064	0.06	0.002
Do not know	0.9	[-9.7, 11.4]	0.872		1.1	[-4.9, 7.1]	0.718	0.01	0.000
Age (per 10 years)	0.2	[-0.7, 1.1]	0.687		-1.3	[-2.2, -0.3]	0.008	-0.13	0.005
Self-rated health	-4.0	[-5.1, -2.8]	< 0.001		-1.5	[-2.3, -0.7]	< 0.001	-0.12	0.010
Mental distress - PHQ4	3.0	[2.7, 3.4]	< 0.001		1.2	[0.9, 1.6]	< 0.001	0.26	0.035
Coping strategies									
Giving up	2.8	[2.3, 3.3]	< 0.001		0.9	[0.5, 1.2]	< 0.001	0.15	0.014
Support	1.4	[1.0, 1.9]	< 0.001		0.6	[0.3, 0.9]	< 0.001	0.13	0.012
Humor	0.4	[-0.4, 1.2]	0.299		-0.7	[-1.2, -0.2]	0.006	-0.08	0.005
Religion	0.3	[-0.5, 1.1]	0.409		0.0	[-0.5, 0.5]	0.972	0.00	0.000
Substance use	4.1	[2.5, 5.7]	< 0.001		1.1	[0.2, 2.0]	0.021	0.07	0.004
**Individual context, care recipient**									
Neuropsychiatric symptoms - NPIQ	1.1	[0.9, 1.2]	< 0.001		0.6	[0.5, 0.7]	< 0.001	0.31	0.067
Dementia severity - IQCODE	8.3	[6.0, 10.7]	< 0.001		2.4	[0.6, 4.3]	0.011	0.09	0.004
Disability - RDRS2	0.5	[0.4, 0.6]	< 0.001		0.2	[0.1, 0.2]	0.001	0.13	0.008
**Relational context**									
ICG primary caregiver: No (ref. Yes)	-6.2	[-10.7, -1.8]	0.006		-4.5	[-7.2, -1.9]	0.001	-0.10	0.008
Relationship: Child or other (ref. Spouse/partner)*	-3.8	[-6.0, -1.6]	0.001						
Cohabiting: No (ref. Yes)	-5.5	[-7.8, -3.3]	< 0.001		-2.2	[-4.7, 0.4]	0.094	-0.09	0.002
Previous relationship satisfaction	-2.6	[-3.8, -1.3]	< 0.001		-1.2	[-1.9, -0.5]	0.001	-0.09	0.007
Extent of care (ref. Once a week or less)									
2–3 times a week	2.6	[-2.2, 7.4]	0.288		2.3	[-0.5, 5.1]	0.102	0.08	0.002
4–6 times a week	4.7	[-0.2, 9.6]	0.061		3.5	[0.7, 6.4]	0.016	0.11	0.004
Every day	10.6	[6.3, 14.8]	< 0.001		4.8	[1.9, 7.7]	0.001	0.20	0.007
**Community context**									
ICG with friends (ref. Weekly)									
Monthly	3.2	[0.6, 5.7]	0.015		0.9	[-0.6, 2.4]	0.245	0.04	0.001
Infrequent	7.1	[4.5, 9.8]	< 0.001		1.7	[0.0, 3.4]	0.052	0.07	0.003
ICG leisure activity	-0.8	[-1.2, -0.5]	< 0.001		0.3	[0.1, 0.6]	0.013	0.08	0.004
CR activity: Any activity (ref. No activity)	-7.0	[-9.7, -4.4]	< 0.001		-1.1	[-2.7, 0.6]	0.206	-0.04	0.001
ICG knowledge of services	-0.8	[-2.0, 0.4]	0.167		-1.1	[-1.8, -0.3]	0.006	-0.09	0.005
ICG social restriction	3.3	[2.5, 4.0]	< 0.001		1.1	[0.6, 1.6]	< 0.001	0.13	0.013
**Time context**									
Caregiving duration (ref. 0-2y)									
3-5y	3.1	[0.6, 5.5]	0.014		0.8	[-0.6, 2.2]	0.268	0.04	0.001
6y or more	3.4	[0.2, 6.7]	0.039		-0.5	[-2.4, 1.4]	0.588	-0.02	0.000
Overall regression statistics									
R2					0.74				
Adjusted R2					0.72				
F (31, 383)					34.62	p < 0.001			

*Strongly correlated with cohabiting variable. sr^2^ = squared semi-partial correlations, ICG = informal caregiver, edu. = educational level, PHQ4 = Patient Health Questionnaire 4, CR = care recipient, NPIQ = Neuropsychiatric Inventory Questionnaire, IQCODE = Informant Questionnaire on Cognitive Decline in the Elderly, RDRS2 = Rapid Disability Rating Scale 2

As shown in Tables 2, 21 out of 26 variables in the univariable models and 17 out of 25 in the multivariable model had statistically significant associations with subjective caregiver burden. This demonstrates that caregiver subjective burden is a complex phenomenon. The following section presents the statistically significant associations with subjective burden in the multivariable model, followed by the most important associations based on relative importance through interpretation of the coefficients, standardized coefficients, and squared semi-partial correlations for each.

In the individual context, informal caregiver factors statistically significantly associated with a higher subjective burden were lower self-rated health, more symptoms of depression and anxiety, using more of the coping strategies resignation and denial (giving up), emotional and instrumental support, and substance use. Older age in informal caregivers and using humor as a coping strategy were associated with a lower subjective burden. Regarding the care recipient, more neuropsychiatric symptoms (NPIQ), more sever dementia (IQCODE), and higher degree of disability (RDRS2) were statistically significantly associated with higher subjective burden.

In the relational context, factors statistically significantly associated with higher burden were increasing extent of care and being the primary caregiver compared to not being the primary caregiver. Being more satisfied with their relationship prior to dementia was associated with a lower subjective burden.

In the community context, factors statistically significantly associated with a higher subjective burden were informal caregivers who had spent time with friends less than monthly compared to weekly, doing more leisure activities, and experiencing higher social restrictions. Informal caregivers having a higher knowledge of the services available for both them and the care recipient were associated with a lower subjective burden.

In the time context, having been an informal caregiver for the care recipient for three years or longer was statistically significantly associated with a higher burden in the univariable model but not in the multivariable model.

Based on interpretation of the coefficients, informal caregivers’ symptoms of depression and anxiety had the greatest relative importance for subjective burden, followed by their coping strategies and self-rated health. Among the care recipient factors, neuropsychiatric symptoms had the highest relative importance. In the relational context, helping the care recipient 4–6 times a week or daily compared to helping the care recipient once a week or less had the highest relative importance. Social restriction had the highest relative importance associated with subjective burden in the community context.

## Discussion

We aimed to explore which individual, relational, community, and time factors are associated with informal caregivers’ subjective burden when caring for home-dwelling individuals with dementia. In the multivariable analysis, statistically significant associations with RSS were found for 17 of the 25 included variables. The multivariable model explained 74% of the variance in subjective caregiver burden in the study population. The most important individual factors related to informal caregivers were mental distress, self-rated health, and coping strategies, while neuropsychiatric symptoms had the strongest association related to the care recipient. In the relationship context, the extent of care and being the primary caregiver had the strongest association with a higher subjective burden. In the community context, being socially restricted due to the caregiver role had the strongest association with an increased burden. In the time context, caregiving durations were only statistically significant in the univariable analyses.

### Associations between subjective burden and contextual factors

#### Individual context - informal caregiver factors

The most important informal caregiver factors were mental distress, coping strategies, and self-rated health. Poorer physical health has previously been linked to higher caregiver burden [55] and more symptoms of depression [56], but the direction of association is unclear. There is a possibility of a bi-directional relationship, where a higher burden might cause deteriorating physical health for the informal caregiver, which makes it challenging to provide care, thereby increasing the subjective burden. Concerning informal caregiver mental distress, more symptoms of depression and anxiety were associated with a higher subjective burden. There was a mean increase of 1.2 units (95% CI 0.9, 1.6) in subjective burden (RSS) per unit increase in the PHQ4 when controlled for the other covariates. Considering the PHQ4 has a range from 0 to 12, this might suggest that it has a substantial effect on subjective burden. However, because we cannot establish causality, there is a possibility that a high subjective burden results in higher mental distress of the informal caregiver. Subjective caregiver burden has been found to be a risk factor for anxiety in informal caregivers [57]. In a longitudinal study, researchers found that poorer mental health among caregivers was associated with increased caregiver burden, and that this burden might be reduced by helping caregivers improve their mental health [55]. Previous studies’ results are inconclusive regarding causality, which might suggest that there is reciprocity between subjective caregiver burden and mental health. Regardless, among the informal caregivers in the present study, 10.6% had a PHQ4 score ≥ 6, indicating moderate to severe mental distress [42]. Therefore, whether it is the burden that gives informal caregivers mental distress or if it is the other way around, informal caregivers should be screened for mental distress and receive help and support in reducing symptoms of depression and anxiety.

Regarding informal caregivers’ coping strategies, using resignation, denial, and substances as coping strategies were associated with a higher subjective burden. These coping strategies are often classified as dysfunctional coping [58], and previous studies have also associated them with increased caregiver burden [21, 59, 60]. Such strategies may negatively affect burden because the informal caregiver ignores or avoids the problem rather than trying to adapt to or accept the situation. Seeking more instrumental and emotional support was associated with higher burden, which was unexpected. Previous studies have found that social and emotion-focused coping strategies may reduce anxiety and depression symptoms [59, 60], which suggests that they could be beneficial for lowering subjective burden. The unexpected findings in the present study may have several reasons. First, seeking others’ support, help, and advice does not always pay off. It depends on whether caregivers receive good support and sound advice. Second, caregivers with higher burden might seek more support, and this negative association could therefore be due to reverse causation. Thus, longitudinal studies are needed to investigate this association further. Using humor as a coping strategy had a small but statistically significant association with lower subjective burden, which is expected. This is an emotion-focused strategy, and the results are in line with previous studies [59, 60]. Conversely, religion is also considered an emotion-focused coping strategy, but we found no evidence of an association between this strategy and subjective burden. This may be due to a weakness in the Norwegian translation of the brief COPE. The Religion factor only includes questions about religion, not spirituality, thus limiting the number of caregivers who report using this coping strategy.

#### Individual context - care recipient factors

For care recipients, an increase in neuropsychiatric symptoms, degree of disability, and worsening of dementia severity were statistically significantly associated with an increased subjective burden. Similar results have been found in previous studies, but with some inconsistencies. All three factors are associated with increased hours of informal care [61], which partly can explain an increased subjective burden. In a review of longitudinal studies, Van Den Kieboom et al. [17] found that both neuropsychiatric symptoms and increased disability were robust risk factors for caregiver burden, while the evidence for cognitive status as a risk factor was not sufficient. Although we found a statistically significant association between subjective burden and dementia severity, this was the care recipient factor with the lowest relative importance. From this we conclude that while dementia severity seems to affect subjective burden for informal caregivers, it is primarily through increased neuropsychiatric symptoms and greater need for help and care.

Concerning neuropsychiatric symptoms, it has also been found that high caregiver burden can lead to more neuropsychiatric symptoms in the person with dementia [15], which again implies a bi-directional relationship between caregiver burden and neuropsychiatric symptoms. A poorer relationship and expressions of irritation or impatience from the informal caregiver can affect the care recipient negatively and worsen neuropsychiatric symptoms [27]. This indicates that an increase in neuropsychiatric symptoms could worsen subjective burden, which could worsen neuropsychiatric symptoms and vice versa, thus creating a vicious spiral that increases the risk of adverse outcomes for both the informal caregiver and the care recipient. This might explain why the association between neuropsychiatric symptoms and subjective burden has the strongest relative importance in the present study. It also emphasizes the importance of informal carers gaining knowledge about neuropsychiatric symptoms and receiving help and support to prevent and manage them.

#### Relationship context factors

Regarding the relationship context, a greater extent of care, being the primary caregiver, and poor previous relationship satisfaction were associated with a higher subjective burden. Primary caregivers reported a higher subjective burden than those who are not, which is expected due to the greater responsibility and care duties. The extent of care’s association with subjective burden is also unsurprising: the more days per week caregivers provide help, the higher the subjective burden. This is in line with previous studies [62, 63]. When the care needs increase or when the person with dementia receives less external informal or formal help, the informal caregiver provides more care. Xu et al. [62] found that caregiver burden was associated with caregiving hours and that the informal caregivers’ social network buffered this association. Considering informal caregivers’ previous relationship satisfaction, the direction of the association with subjective burden showed that the more satisfied the informal caregiver was with the relationship before the onset of dementia, the lower the subjective burden. These results align with previous studies [27, 64]. The reason for this association may be that in an already strained relationship, there is less tolerance or resilience in responding to the challenges and worries experienced by the caregiver.

In the present study, the correlation between cohabitation status and relationship type (spouse or other) was too high to include both in the multivariable model. This was because almost all informal caregivers who lived with the care recipient were spouses, and those not living with the care recipient had other relations. Thus, we found no statistically significant associations between subjective burden and these variables in the multivariable model. Viñas-Diez et al. [65] found that caregiver burden varied depending on the relationship and cohabitation status between caregivers and individuals with Alzheimer’s disease. In their study, spouses had a medium burden that gradually increased, while cohabiting children had a stable high burden and non-cohabiting children had a stable but lower burden than the other two groups. However, we can neither confirm nor deny these results due to the present study’s sample distribution.

#### Community context factors

In the community context, informal caregivers feeling socially restricted by their caregiving role, seeing friends less often, doing more leisure activities, and having less knowledge of available services were statistically significantly associated with increased subjective burden. Being socially restricted had the strongest association with an increased burden, according to the standardized coefficient and squared semi-partial correlations. The association between subjective burden and social restriction is supported by previous studies showing that social support tends to lower the burden [28, 63] while social isolation increases the burden [27]. These previous findings are also in line with the present study’s results regarding how often the informal caregiver spends time with friends. The association between informal caregivers’ leisure activities and subjective burden changes direction between the univariable and multivariable models. Univariably, it appears that leisure activities can lower subjective burden because those who are able to participate in many activities have care recipients who are healthier. When controlling for the other factors, those participating in more activities have a higher burden. This result from the multivariable model may be due to feelings of guilt. Informal caregivers may have conflicting feelings between prioritizing spending time with the care recipients and prioritizing self-care, which can lead to feelings of guilt and thus to increased subjective burden [66]. Regarding informal caregivers’ knowledge of available services, having higher knowledge was associated with a lower burden. Henriksen et al. [67] found that informal caregivers with poor information on available services were more likely to experience care as demanding, but the authors could not rule out reverse causation. We agree that this association can go both ways. We believe that informal caregivers who know of the available services can feel less stressed because they know what kind of help they can receive and may better utilize the services. However, there is a possibility that relatives who experience a greater burden may have fewer resources to familiarize themselves with available services. Longitudinal studies are needed to further explore this.

#### Time context - caregiving duration

Caregiving duration was only statistically significant in the univariable analyses. The burden reasonably increases over time due to disease progression, but when controlling for factors such as care recipients’ neuropsychiatric symptoms and disability, time in itself does not have an effect. The trajectory of dementia varies from person to person, in terms of both the speed of deterioration of cognitive and physical functions and the presence of symptoms. Thus, how long the informal caregiver has been caring for the care recipient does not alone explain burden. This is evident in the results reported by Conde-Sala et al. [55], who found three different trajectories of burden in a sample of informal caregivers over a period of three years: one group with a relatively stable low burden, one group where burden increased over time, and one group where burden decreased over time. The three groups differed in care recipients’ neuropsychiatric symptoms and caregivers’ mental health. Although we cannot rule out that time might have an effect on subjective burden, the results from the present study and from Conde-Sala et al. suggest that individual factors related to the caregiver and care recipient are more important.

#### Most important factors

Of all factors, care recipients’ neuropsychiatric symptoms and informal caregivers’ symptoms of depression and anxiety are the two covariates with the strongest associations to subjective caregiver burden based on an overall assessment of coefficients, standardized coefficients, and squared semi-partial correlations. Based on these findings, we believe a particular focus on these two factors is important to best address subjective burden. We do not think it is realistic to eradicate subjective burden, but relieving subjective burden could benefit both informal caregivers and care recipients and postpone the need for institutional care. Healthcare personnel should screen for care recipients’ neuropsychiatric symptoms and informal caregivers’ mental distress, in addition to screening for subjective burden, to reveal the need for interventions. Because of the potential bi-directionality in the associations between subjective burden and these two factors, improving either one might result in improvements in the others, as previously stated by Isik et al. [27].

A large variety of interventions have shown promising results, including various types of psychosocial and psychoeducational interventions that might improve care recipients’ neuropsychiatric symptoms and informal caregivers’ well-being and coping [60] and reduce informal caregivers’ depression and anxiety [68]. However, the evidence is inconsistent, and researchers have called for interventions adapted to subgroups of informal caregiver and care recipient dyads [69]. In line with our results, we suggest that interventions aimed toward informal caregivers and people with dementia should be adapted to the specific needs of each dyad’s individual and contextual factors.

### Strengths and limitations

The present study included a large sample of geographically and demographically diverse informal caregivers of individuals with dementia at different stages of the disease. We have also incorporated a broad range of covariates, including internationally accepted validated questionnaires for key concepts. Although a large number of covariates may increase the risk of overfitting the model, we consider this broad range of covariates a strength in the present study due to the large number of participants and because the covariates are selected based on previous research and theory. The study has several limitations that should be considered. First, the study was cross-sectional, so the direction of causation is not possible to determine. Second, we have used a convenience sample of informal caregivers. We have tried to mitigate this with a broad recruitment strategy and a large sample. Also, the study was conducted only in Norway. Previous studies have found cultural and societal differences in the experience of caregiving [70, 71]. Therefore, caution should be made regarding generalization, especially across cultures and nations. Third, the study was conducted during the COVID-19 pandemic. Studies from the first months of the pandemic in Norway showed an increase in informal care hours [72] and a worsening of neuropsychiatric symptoms [73] that might have affected subjective burden. Due to variations in national and local restrictions in 2021, it is difficult to evaluate the impact. Although burden and neuropsychiatric symptoms might have been higher due to the pandemic, we believe it has not significantly affected the associations between subjective burden, neuropsychiatric symptoms, and the covariates. Fourth, the study is based solely on informal caregivers’ self-reports, so care recipient covariates such as neuropsychiatric symptoms and dementia severity has not been validated by healthcare personnel. We have tried to minimize possible bias by using questionnaires adapted and validated for informal caregivers. Fifth, since subjective burden of care is a complex, subjective, and abstract concept, many factors come into play. For example, positive aspects of caregiving [63, 74, 75] and personality traits [76, 77] have received much attention in recent studies. We have not collected data on these factors in our study, and future studies or meta-analyses should investigate how these factors may affect other individual and contextual factors.

## Conclusions

In this study, we found evidence of associations between several individual and contextual factors and subjective burden in informal caregivers. The fact that many factors seem to be associated with care burden emphasizes the complexity of the phenomenon. Informal caregivers’ mental distress and care recipients’ neuropsychiatric symptoms were the factors with the strongest association with subjective burden. Previous studies have indicated possible bi-directionality in these associations. Therefore, neuropsychiatric symptoms, informal caregiver mental distress, and subjective burden should be screened for by healthcare personnel and targeted in interventions. Longitudinal studies are needed to explore causality further and future studies should also emphasize service provision and factors at the societal level.

### Electronic supplementary material

Below is the link to the electronic supplementary material.

Supplementary Material 1

Supplementary Material 2

## Data Availability

The data presented in this study might be provided by the corresponding author if granted approval from the Regional Committee for Medical Research Ethics Central Norway. The data are not publicly available due to ethical restrictions. Due to the nature of this research, participants of this study were not asked to agree for their data to be shared publicly.
