# Supplementary material for: Factors associated with subjective burden among informal caregivers of home-dwelling people with dementia: a cross-sectional study
Source: BMC Geriatr. 2023 Oct 10;23:644. doi: 10.1186/s12877-023-04358-3 (PMC10565959; doi:10.1186/s12877-023-04358-3)
Supplement: Supplementary file 1 — Supplementary Material 1 [file 12877_2023_4358_MOESM1_ESM.docx]

## Supplementary 2. Standardized residuals of multivariable linear regression

**Supplementary figure 1**. Histogram of standardized residuals after multivariable linear regression.

**Supplementary figure 2**. Quantiles of standardized residuals after multivariable linear regression against quantiles of the standard normal distribution.

**Supplementary figure 3**. Scatterplot of standardized residuals and predicted RSS. Breusch–Pagan test for heteroskedasticity for fitted values of RSS not significant (p= 0.71).
