# Supplementary material for: Factors associated with subjective burden among informal caregivers of home-dwelling people with dementia: a cross-sectional study
Source: BMC Geriatr. 2023 Oct 10;23:644. doi: 10.1186/s12877-023-04358-3 (PMC10565959; doi:10.1186/s12877-023-04358-3)
Supplement: Supplementary file 2 — Supplementary Material 2 [file 12877_2023_4358_MOESM2_ESM.docx]

## Supplementary 1. Exploratory factor analyses of the brief COPE

**Table S1** Exploratory factor analyses of the brief COPE.

| Variable | F1 | F2 | F3 | F4 | F5 | F6 | F7 | F8 | Uniq |
| --- | --- | --- | --- | --- | --- | --- | --- | --- | --- |
| 1. Turning to other activities to take my mind off it | 0.77 |  |  |  |  |  |  |  | 0.43 |
| 2. Concentrating on doing something about the situation | 0.82 |  |  |  |  |  |  |  | 0.41 |
| 3. Saying to myself "this isn't real" |  |  | 0.61 |  |  |  |  |  | 0.46 |
| 4. Using alcohol or other drugs to make myself feel better |  |  |  |  | 0.96 |  |  |  | 0.12 |
| 5. Getting emotional support from others |  | 0.80 |  |  |  |  |  |  | 0.33 |
| 6. Giving up trying to deal with it |  |  | 0.87 |  |  |  |  |  | 0.33 |
| 7. Taking action to try to make the situation better | 0.67 |  |  |  |  |  |  |  | 0.45 |
| 8. Refusing to believe that it has happened |  |  | 0.77 |  |  |  |  | 0.31 | 0.33 |
| 9. Saying things to let my unpleasant feelings escape |  |  |  |  |  |  |  |  | 0.57 |
| 10. Getting help and advice from other people |  | 0.85 |  |  |  |  |  |  | 0.28 |
| 11. Using alcohol/other drugs to help me get through it |  |  |  |  | 0.96 |  |  |  | 0.12 |
| 12. Trying to see it in a different light, to make it seem more positive |  |  |  |  |  |  |  | 0.47 | 0.52 |
| 13. Criticizing myself |  |  |  |  |  |  | -0.46 |  | 0.56 |
| 14. Trying to come up with a strategy about what to do | 0.71 |  |  |  |  |  |  |  | 0.47 |
| 15. Getting comfort and understanding from someone |  | 0.89 |  |  |  |  |  |  | 0.23 |
| 16. Giving up the attempt to cope |  |  | 0.86 |  |  |  |  |  | 0.32 |
| 17. Looking for something good in what has happed |  |  |  | 0.36 |  |  |  | 0.51 | 0.51 |
| 18. Trying to make jokes about it |  |  |  | 0.88 |  |  |  |  | 0.24 |
| 19. Doing something to think less about it (cinema, watching TV, reading, sleeping, etc.) | 0.59 |  |  |  |  |  |  |  | 0.49 |
| 20. Accepting the reality of the fact that it has happened. | 0.41 |  |  |  |  |  | 0.59 |  | 0.48 |
| 21. Expressing my negative feelings | 0.51 |  |  |  |  |  |  | -0.42 | 0.48 |
| 22. Trying to find comfort in my faith or religion. |  |  |  |  |  | 0.95 |  |  | 0.10 |
| 23. Trying to get advice or help from other people about what to do |  | 0.65 |  |  |  |  |  |  | 0.43 |
| 24. Learning to live with it. |  |  |  |  |  |  | 0.60 | 0.43 | 0.45 |
| 25. Thinking hard about what steps to take. | 0.35 |  |  |  |  |  |  | 0.56 | 0.47 |
| 26. Blaming myself for things that happened |  |  |  |  |  |  | -0.65 |  | 0.54 |
| 27. Praying to God for help. |  |  |  |  |  | 0.96 |  |  | 0.10 |
| 28. Trying to make fun of the situation. |  |  |  | 0.91 |  |  |  |  | 0.24 |

PCF with promax rotation. F=factor, F2 = “Support”, F3 = “Giving up”, F4 = “Humor”, F5 = “Substance use”, F6 = “Religion”, Uniq = uniqueness.
